# Evaluation de la performance du système de gestion logistique des intrants de lutte contre le paludisme dans le Département du Littoral, au Bénin, en 2017

**DOI:** 10.11604/pamj.2018.29.61.14024

**Published:** 2018-01-22

**Authors:** Abdou-Rahim Ouro-Koura, Emmanuel Ghislain Sopoh, Jerôme Charles Sossa, Yolaine Glèlè-Ahanhanzo, Victoire Agueh, Edgard-Marius Ouendo, Laurent Ouedraogo

**Affiliations:** 1 Institut Régional de Santé Publique Comlan Alfred QUENUM (IRSP CAQ), Ouidah, Bénin; 2 Centre de Formation en Santé Publique (CFSP) de Lomé, Togo

**Keywords:** Performance, gestion logistique, paludisme, Littoral, Bénin, Performance, logistics management, malaria, Littoral, Benin

## Abstract

**Introduction:**

Cette étude visait à évaluer la performance du système de gestion logistique (SGL) des intrants de lutte contre le paludisme (ILP) dans le Département du Littoral, au Bénin, en 2017.

**Méthodes:**

Il s’agissait d’une étude transversale évaluative qui s’était déroulée en juin 2017. Elle portait sur les structures de stockage et de cession des ILP ainsi que le personnel impliqué dans leur gestion. La performance du SGL était évaluée à partir de la conformité observée pour les composantes et sous-composantes de la « Structure », du « Processus » et des « Résultats » par rapport aux normes ou standards définis par le Ministère de la Santé.

**Résultats:**

Un total de 36 structures a été enquêté avec leurs cibles secondaires. Il résulte que 52,78% des dépôts répartiteurs réunissaient les conditions optimales de stockage des intrants alors que seulement 33,33% des agents chargés de la gestion des ILP étaient formés en gestion logistique. La performance du SGL des ILP était insuffisante (conformité de 59,13% par rapport au score attendu). La structure, ainsi que le processus avaient une conformité Insuffisante par rapport aux normes (respectivement 60,20% et 73,22% du score attendu), engendrant des résultats jugés mauvais (41,53% du score attendu). La sous-composante la plus insuffisante était le système d’information de la gestion logistique (SIGL).

**Conclusion:**

Cette étude met en évidence la place du SIGL pour une meilleure performance de la gestion des ILP. Une attention particulière devra être accordée à ce volet.

## Abstract (en)

**Introduction:**

This study aimed to evaluate the performance of the logistics management system (LMS) of malaria control (MC) resources in the Littoral Department, Benin, in 2017.

**Methods:**

In June 2017, we conducted a cross-sectional evaluative study focusing on the structures for the storage and the disposal of MC resources as well as on staff involved in their management. The performance of the the logistics management system was evaluated on the basis of the observed compliance of the components and sub-components of the “Structure”, the “Process” and the “Results” with the norms and standards defined by the Ministry of Health.

**Results:**

A total of 36 structures were investigated and secondary target was surveyed. It followed that 52,78% of the structures for the storage and the disposal of MC resources met the requirements for resources storage while only 33.33% of MC resources management staff were trained in logistics management. The performance of the logistics management system of MC resources was inadequate (compliance 59,13 % compared to the expected score). The structure, as well as the process were non-compliant with the standards ( 60,20% and 73.22% compared to the expected score respectively), leading to negative results (41.53% compared to the expected score). The most inadequate sub-component was the logistics management information system (LMIS).

**Conclusion:**

This study highlights the role of LMS for better performance of MC resources management. Particular attention should be given to this component.

## Introduction

Le paludisme est la maladie parasitaire la plus répandue dans le monde [1]. En 2016, l’Organisation Mondiale de la Santé (OMS) estimait à 3,2 milliards le nombre de personnes, exposées au risque de paludisme. En 2015, 90% des cas de paludisme et 92% des décès étaient survenus dans 13 pays d’Afrique. Les enfants âgés de moins de cinq ans et les femmes enceintes sont les plus atteints. Le paludisme tue entre 1,1 et 2,7 millions de personnes dans le monde chaque année, dont environ un million d’enfants de moins de cinq ans résidant en Afrique subsaharienne [2]. Au Bénin, le paludisme demeure un problème de santé publique avec une incidence élevée. Selon l’annuaire des statistiques sanitaires [3] il était la première cause d’hospitalisation en 2013 avec 29,2% des cas enregistrés. Une des stratégies retenues dans la lutte contre le paludisme est le diagnostic et le traitement des cas, d’où la nécessité pour les pays de s’approvisionner en intrants, gage de l’offre de soins de qualité aux populations à tous les niveaux. Au rang des mesures prises en matière de lutte contre le paludisme figure, entre autres, la gratuité de la prise en charge des cas chez les enfants de moins de cinq ans et chez les femmes enceintes [4]. Le Bénin bénéficie de l’appui de plusieurs partenaires pour son approvisionnement en Intrants de Lutte contre le Paludisme (ILP). Depuis 2014, différents documents du programme révèlent des faiblesses du SGL des ILP qui se caractérisent par des ruptures de stocks, des sur-stockages et des péremptions des différents intrants à tous les niveaux du système [5-7]. Plusieurs raisons pourraient expliquer ces faiblesses, notamment les insuffisances dans les données rendues disponibles par le système d’information de la gestion logistique (SIGL). Le Département du Littoral, à l’instar des autres départements du pays est concerné par le phénomène. En vue de contribuer à l’amélioration de la disponibilité des ILP, nous nous sommes intéressés à l’organisation et au fonctionnement de toute la chaîne de la gestion logistique dans ce département.

## Méthodes

**Type d’étude**: Il s’agit d’une étude transversale évaluative qui s’est déroulée dans le département du Littoral du 6 au 29 juin 2017.

**Population d’étude**: Les cibles primaires de notre étude étaient constituées par les dépôts de pharmacie (dépôts répartiteurs de zones, entrepôts des formations sanitaires (FS) et points de cession des ILP) des FS publiques et privées du département du Littoral. Les Cibles secondaires étaient: les gestionnaires des dépôts répartiteurs de zones (GDRZ), les responsables des entrepôts des FS, les commis à la vente des médicaments (au point de cession), les bénéficiaires des ILP, les responsables de FS et le chef du service de pharmacie et pharmacovigilance du programme national de lutte contre le paludisme (PNLP).

**Echantillonnage**: Trente-deux FS ont été sélectionnés par tirage aléatoire simple, sur les 52 du département. Les responsables de ces FS, ainsi que les responsables de dépôts et commis des postes de cession ont été enquêtés. De même, les dépôts répartiteurs des quatre zones sanitaires (ZS) du département, ainsi que leurs responsables ont été également enquêtés. Un bénéficiaire, identifié par commodité, a été enquêté dans chaque FS.

### Variables

**Figure 1:**
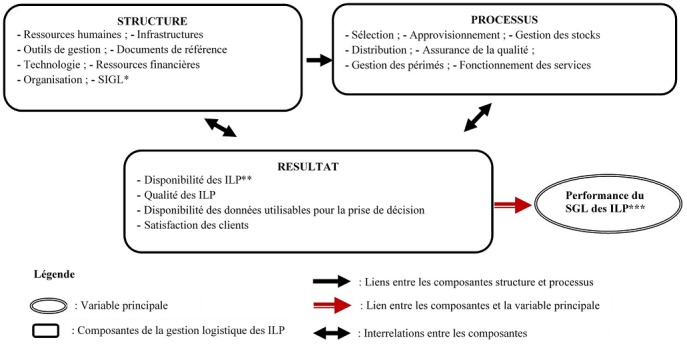
Cadre conceptuel de l’évaluation de la performance du SGL des ILP dans le département du Littoral en 2017, adapté du modèle logique d’intervention de Donabedian; SIGL = Système d'Information de Gestion Logistique; SGL = Système de Gestion Logistique; ILP= Intrants de Lutte contre le Paludisme

La performance du SGL des intrants de lutte contre le paludisme a été appréciée sur la base de la combinaison des scores de conformité de la structure, du processus gestionnaire et des résultats, par rapport aux normes du Ministère de la Santé [3, 7]. La structure a été appréciée sur la conformité aux normes, des ressources humaines, ressources financières, infrastructures, outils de gestion, documents de référence, de la technologie et du système d’information de la gestion logistique. Le processus gestionnaire a été apprécié sur la conformité aux normes de la sélection, l’approvisionnement, la gestion des stocks, la distribution, l’assurance de la qualité, la gestion des périmés et le fonctionnement des services. Quant aux résultats, ils ont été appréciés sur la conformité aux normes des indicateurs clé comme la disponibilité des ILP, la qualité des ILP, la disponibilité des données du SIGL pour la prise de décisions, la satisfaction des clients internes et ceux externes. La Figure 1 présente le modèle théorique utilisé, adapté du modèle logique d’intervention de Donabedian [8] (Figure 1). Chaque item évalué a été côté 1 lorsqu’il est conforme à la norme nationale, et 0 lorsqu’il ne l’est pas. La compilation des scores pour les trente-six structures évaluées nous donne le score total attendu par sous-composante et par composante. La proportion des conformités par rapport aux scores attendus donne le taux de conformité pour chaque sous-composante et composante. La performance globale du système est la proportion du score total obtenu par rapport au score total attendu pour les trois composantes. Le Tableau 1 résume les scores attendus par composante et sous-composante. De façon empirique, nous avons retenu que: lorsque la conformité aux normes est supérieure ou égale à 80%, cela signifie que la quasi-totalité des critères d’appréciation sont satisfaisants. La performance de la composante est jugée « Bonne »; Lorsque la conformité aux normes est comprise entre 50 et 80%, cela signifie que peu de critères d’appréciation sont satisfaisants. La performance de la composante est jugée « Insuffisante »; Lorsque la conformité aux normes est inférieure à 50%, cela signifie que très peu des critères d’appréciation sont satisfaisants. L’appréciation de la performance de la composante est jugée « Mauvaise » (Tableau 1).

**Tableau 1:** Récapitulatif de l’aspect opérationnel des variables par composantes et sous-composantes, selon les scores et appréciations de la performance du SGL des ILP dans le département du Littoral en 2017

Composantes	Sous-composantes	Score attendu
**Structure**		
	Ressources humaines	324
	Infrastructure	432
	Outils de gestion	316
	Technologie	16
	Ressources financières	180
	SIGL	444
**Total structure**		**1712**
**Processus**		
	Sélection	36
	Quantification	180
	Stockage	498
	Gestion des ILP périmés	72
	Distribution/Cession des ILP	160
	Assurance qualité des ILP	108
	Commande et réception	216
	Fonctionnement des services	120
**Total Processus**		**1390**
**Résultat**		
	Disponibilité des ILP	1296
	Disponibilité des données du SIGL pour la prise de décisions	36
	Satisfaction des clients	168
**Total résultat**		**1500**

### Techniques et outils de collecte des données

Les données ont été recueillies par des enquêteurs formés. Les outils utilisés sont: un questionnaire pour les gestionnaires de DRZ, responsables d’entrepôts de FS, les commis à la vente et clients externes; un guide d’entretien pour les responsables de FS et le chef du service de pharmacie et pharmacovigilance du PNLP; une fiche de dépouillement des outils de gestion; une grille d’observation des magasins et entrepôts. Les outils ont été pré-testés dans deux FS du département de l’Atlantique avant l’enquête, puis corrigés au besoin.

### Traitement et analyse des données

Les données ont été saisies à l’aide du logiciel Excel^®^ et analysées avec le logiciel STATA.11. Les proportions de conformité par rapport aux normes ont été calculées globalement pour les 36 structures évaluées, par sous-composante, puis par composante.

### Considérations éthiques et déontologiques

L’enquête a été autorisée par le Ministère de la santé et les responsables départementaux ainsi que ceux de chacune des FS. Le consentement oral des participants a été obtenu avant leur implication dans l’enquête. Leur anonymat a été conservé lors du traitement des données.

## Résultats

### Description de l’échantillon d’étude

L’ensemble des 36 structures prévues ont été enquêtées, dont quatre dépôts répartiteurs, 32 entrepôts de FS, dont 12 publics, 18 privés et deux confessionnels. Par ailleurs, 32 responsables de FS, 32 commis à la vente des ILP et autres intrants, 01 chef du service de pharmacie et pharmacovigilance du PNLP et 32 bénéficiaires des ILP dans les FS ont été enquêtés.

### Description des composantes structure, processus, résultats du SGL des ILP dans le département du Littoral en 2017

### Composante structure

### Ressources humaines

*Disponibilité des ressources humaines*: chaque DRZ est géré par un comptable qui assure aussi la comptabilité du bureau de zone. Au niveau des entrepôts des FS, on notait une à trois personnes chargée(s) de la gestion des médicaments et ILP. Les profils des responsables se présentent comme suit: 14 comptables (43,75%); 9 aide soignants (28,13%); 6 Infirmiers (18,75%). Les autres (magasinier, secrétaire et informaticien) sont 3 et représentent 9,37%. Le nombre d’années médian d’expérience des agents dans la gestion des dépôts est de 5 ans (3,5 ans; 8 ans). Dix agents (dont 8 du secteur public) sur les 36, soit 27,78% étaient à leur poste il y a moins de 3 ans.

*Formation du personnel en gestion logistique*: des 36 agents de dépôts pharmaceutiques, 12 (dont 2 Gestionnaires de DRZ), soit 33,33% ont bénéficié d’une formation en gestion logistique des ILP. Parmi eux, 4 ont bénéficié d’une remise à niveau au cours des 3 dernières années. Il n’y a pas eu de nouvelle formation au cours de 3 dernières années.

*Supervision*: des 36 agents enquêtés, 31, soit 86,11% ont déclaré avoir été supervisés au moins une fois au cours des trois derniers mois par leur hiérarchie. Les équipes de supervision venaient de la direction départementale de la santé (DDS), du PNLP ou parfois étaient des équipes conjointes.

**Infrastructures et moyens de transport des ILP**: Dix-neuf structures (2 DRZ et 17 entrepôts de FS) sur les 36, soit 52,78% disposant de magasin réunissent au moins 80% des conditions optimales pour le stockage des ILP. Parmi ces dernières, on retrouve seulement cinq FS privées sur les 18 enquêtées.

**Outils de gestion de stocks des ILP**: Vingt-trois (23) structures sur les 36, soit 63,89% disposaient de 100% des fiches de gestion de stock des 12 ILP à l’étude. Le remplissage des fiches était à jour dans 47,22% des cas, soit 17 structures sur les 36.

### Système d’information de la gestion logistique

Trois DRZ sur les quatre et 8 entrepôts de FS sur les 32 enquêtés (25%) avaient transmis les rapports des 6 derniers mois en respectant le délai et le rythme (la promptitude). La complétude des rapports des FS au niveau des DRZ pour les 6 derniers mois était de 67,91% soit 67 rapports reçus sur 110 attendus. Dans 21 structures sur 36, soit 58,33% on notait une discordance entre les données du rapport et celles de l’inventaire physique au moment de l’élaboration des rapport/commandes. Le logiciel de gestion (MEDISTOCK^®^) était disponible au niveau des quatre DRZ. Trois GDRZ sur 4 étaient formés à son utilisation. Il était fonctionnel et utilisé pour les activités dans deux DRZ sur les 4.

### Composante processus gestionnaire

### Sélection, quantification, commande/réception

La sélection des ILP est faite par le niveau central; 55,56% (20 sur 36) des structures disposaient de la liste des ILP retenus par la politique nationale et s’y réfèrent pour les commandes. La quantification des besoins en ILP se fait à chaque niveau de la pyramide et 77,78 % des responsables de la gestion des ILP en sont responsables. Les niveaux minimum et maximum des différents ILP ont été définis dans 14 structures sur les 36 enquêtées, soit 38,89%. Selon les GDRZ et des entrepôts des FS la faible disponibilité des intrants au niveau central limite leur application.

### Conditions de stockage des ILP

Nous avons trouvé que 66,67% des structures réunissaient 80% et plus de critères normatifs de stockage des ILP dans les magasins. Toutes les structures (100%) respectent le stockage des périmés mais 31 sur 36, soit 86,11% avaient encore des intrants périmés dans les FS.

### Composantes résultats

**Figure 2:**
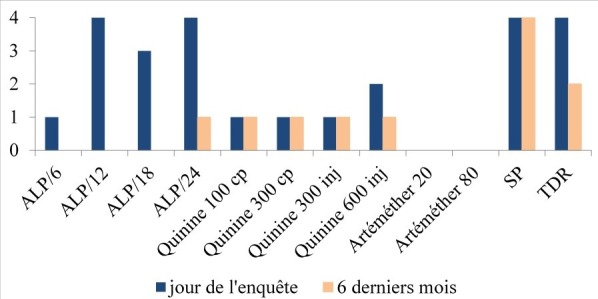
Nombre de DRZ disposant d’ILP le jour de l’enquête et au cours des 6 derniers mois, dans le département du Littoral en 2017; ALP = Arthéméter-Luméfantrine Plaquette de 6,12, 18 ou 24; SP= Sulfadoxine-Pyriméthamine; TDR= Test de Dépistage Rapide

La disponibilité des ILP a été appréciée sur douze intrants traceurs. Dans les DR, la disponibilité des ILP dans les DRZ le jour de l’enquête et au cours des 6 derniers mois est résumé sur la Figure 2. Le jour de l’enquête, aucun DR ne disposait de tous les ILP traceurs appréciés. Trois DR disposaient seulement d’un ILP et 1 DR disposait de 4 ILP. Dans les six mois précédant l’enquête, aucun DR ne disposait de tous les ILP traceurs appréciés. Quatre DR disposaient de la Sulfadoxine-Pyriméthamine (SP) et deux des tests de diagnostic rapide. Un seul DR disposait de cinq ILP (Figure 2).

### Disponibilité des intrants

Au cours des six derniers mois, le taux de disponibilité des ILP dans les DRZ variait de 0% (pour l’Artéméther 20 et 80 mg) à 100% (pour la SP). Dans les FS ce taux variait entre 11,70% pour Artéméther 20 mg et 86,36% pour la Quinine injectable 600 mg (Tableau 2).

**Tableau 2:** Taux de disponibilité des ILP^+^ dans les DRZ^++^ et dans les FS^+++^ au cours des 6 derniers mois dans le département du Littoral en 2017

ILP	Taux de disponibilité	
	DRZ (%)	FS (%)
ALP^¥^/6	51,80	35,38
ALP/12	78,92	30,58
ALP/18	42,32	26,37
ALP/24	66,18	39,34
Quinine 100 comprimé	35,78	65,69
Quinine 300 comprimé	36,44	71,08
Quinine 300 injectable	25,00	52,86
**Quinine 600 injectable**	25,00	**86,36**
**Artéméther 20**	**0,00**	11,70
**Artéméther 80**	**0,00**	47,65
**SP^¥^**	**100,00**	58,68
TDR^¥¥^	93,63	43,97

+ ILP: Intrants de Lutte contre le Paludisme

++ DRZ: Dépot Répartiteur de Zone

+++ FS: Formation Sanitaire

¥ ALP: Artémether-Luméfantrine Plaquette

¥¥ SP: Sulfadoxine-Pyrimétamine

¥¥¥ TDR: Test de Dépistage Rapide

### Satisfaction des clients

Un gestionnaire de DRZ sur les 4 et 28,12% des responsables d’entrepôts de FS ont déclaré avoir été satisfaits de la livraison des quantités d’ILP commandées. Sur 32 clients externes interrogés, 22 ont déclaré être satisfaits de la disponibilité des ILP dans les FS ; ils représentent 68,75%. Vingt et neuf d’entre eux, soit 90,63% trouvaient que les prix des intrants vendus étaient supportables.

### Appréciation du niveau de performance du SGL des ILP dans le département du Littoral en 2017

La conformité des sous-composantes de la « Structure » variait de 47,30% pour le SIGL, à 76,85% pour « l’infrastructure et les moyens de transport ». La conformité de la sous-composante « Technologie » était à 75%; celle des « outils de gestion », des « ressources financières » et des « Ressources Humaines » étaient respectivement de 73,73%, 68,33%, et 53,09%. L’appréciation de la performance de la composante « Structure » est insuffisante avec conformité de 63,20% du total attendu (Tableau 3). La conformité des sous-composantes du « Processus » variait de 50, 63% pour la « Distribution/cession des ILP », à 93,52% pour « l’Assurance Qualité des ILP ». La conformité des autres sous-composantes était respectivement de 55,56% (Sélection), 56,94% (Gestion des périmés), 65,74% (Commande et réception), 66,67% (Quantification), 82,33% (Stockage) et 87,04% « Fonctionnement des services). Le Processus est dans son ensemble jugé insuffisant avec 73,22% de conformité (Tableau 3). Les conformités des sous-composantes du « Résultat » étaient respectivement, de 40,43% (Disponibilité des ILP), 41,67% (Disponibilité des données pour la prise de décision) et 50,00% (Satisfaction du client). Les résultats sont mauvais avec 41,53% de score total attendu (Tableau 4). Au total, la performance du SGL des ILP dans le département du Littoral est insuffisante, avec une conformité de 59,13% par rapport aux normes (Tableau 4).

**Tableau 3:** Appréciation des composantes « Structure », Processus et « Résultats » et de leurs sous-composantes pour l’évaluation de la performance du SGL^+^ des ILP^++^ dans le Littoral en 2017

Composantes et Sous-composantes	Score attendu	Score obtenu	Conformité (%)
Structure			
Ressources Humaines	324	172	53,09
Infrastructure et moyen de transport	432	332	76,85
Outils de gestion	316	233	73,73
Technologie	16	12	75,00
Ressources financières	180	123	68,33
Système d'Information de la Gestion Logistique	444	210	47,30
Total structure	1712	1082	63,20
Processus			
Sélection	36	20	55,56
Quantification	180	120	66,67
Commande et Réception	216	142	65,74
Stockage	498	410	82,33
Distribution/Cession des ILP	160	81	50,63
Assurance Qualité des ILP	108	101	93,52
Gestion des ILP périmés	72	41	56,94
Fonctionnement des services	108	94	87,04
Total processus	1378	1009	73,22
Résultats			
Disponibilité des ILP	1296	524	40,43
Disponibilité des données pour la prise de décisions	36	15	41,67
Satisfaction des clients	168	84	50,00
Total résultats	1500	623	41,53

+ SGL: Système de Gestion Logistique

++ ILP: Intrants de Lutte contre le Paludisme

**Tableau 4:** Appréciation globale des composantes de la performance du SGL^+^ des ILP^++^ dans le département du Littoral en 2017

Composantes	Score attendu	Score obtenu	Conformité (%)	Appréciation de la performance
Structure	1712	1082	63,20	Insuffisante
Processus	1378	1009	73,22	Insuffisante
Resultats	1500	623	**41,53**	**Mauvaise**
**Performance du SGL**	**9180**	**5428**	**59,13**	**Insuffisante**

+ SGL: Système de Gestion Logistique

++ ILP: Intrants de Lutte contre le Paludisme

## Discussion

Cette étude a permis d’apprécier le niveau de performance du SGL des ILP dans le département du Littoral en 2017. Elle a identifié comme point de faiblesse majeur, l’insuffisance de mise en œuvre du SIGL. Les 36 structures (32 entrepôts de FS et 4 DRZ) prévus ont été investigués, avec leurs cibles secondaires. Elles représentent 60% des FS du département. Les 32 entrepôts, sélectionnés de manière aléatoire, regroupaient aussi bien des FS publiques que privées. La méthode d’échantillonnage ainsi que l’effectif des cibles, garantissent donc la représentativité des résultats à l’échelle du département.

### Ressources humaines

La sous-composante ressources humaines a été appréciée « Insuffisante » avec 53,09% du score attendu, compte tenu des insuffisances notées en termes d’effectif (surtout au niveau des DRZ), de qualification, de formation et d’organisation. Au niveau des DRZ il n’y avait qu’un seul agent, le comptable, ayant par ailleurs d’autres charges au bureau de zone, alors qu’il devrait être secondé par un magasinier ou un logisticien. Seulement 33,33% des agents ont bénéficié d’une formation en gestion logistique des ILP. Cette proportion est en deçà des résultats obtenus par Kolapo et al. [9] en 2007 au Nigéria, lors d’une évaluation du SGL des produits contraceptifs ; dans cette étude, 95% du personnel impliqué dans la gestion logistique des contraceptifs étaient formés. La faible proportion du personnel formé dans le Littoral pourrait s’expliquer par les affectations du personnel et la proportion élevée des structures privées. En effet, 10 agents (dont 8 du secteur public) sur les 36 (soit 27,78%) occupent leur poste actuel depuis moins de 3 ans, alors qu’il n’y a pas eu de formation de gestionnaires des dépôts pharmaceutiques en gestion logistique au cours de cette période. Dans notre étude, 86% du personnel avait bénéficié d’au moins une supervision au cours des trois derniers mois. Ce résultat est similaire à ceux de Ouédraogo et al en 2006 [10] en République Démocratique du Congo (RDC) (75% du personnel supervisés surla gestion des produits) et de loin meilleur à ceux relevés par Kolapo et al. [9] (seulement 29% du personnel impliqué supervisés). Au total, si la fréquence des supervisions du personnel chargé de la gestion logistique des ILP parait satisfaisante, il reste à améliorer l’effectif (surtout dans les DRZ) et la formation ou remise à niveau des agents.

### Infrastructures et conditions de stockage des intrants

Il résulte de l’étude que 52,78% des structures enquêtées disposaient de magasin réunissant au moins 80% des conditions optimales pour le stockage des ILP et autres intrants/médicaments. En RDC, 64,2% la proportion des FS remplissait au moins 80% des conditions de stockage de produits contraceptifs [10]. Au Nigéria, 70% des entrepôts évalués réunissaient ces conditions [9]. Par contre au Soudan du Sud en 2011 [11] seulement 35% des magasins et installations respectaient les conditions acceptables de stockage des produits. Selon les auteurs, ce résultat pourrait s’expliquer par l’immaturité du système Sud Soudanais à appuyer les structures pour mettre en place des dépôts pharmaceutiques. Notre résultat peut s’expliquer par le fait que la plupart des magasins ne réunissant pas les conditions satisfaisantes de stockage des intrants étaient retrouvés dans les structures privées (12 FS privées sur les 17 concernées). L’insuffisance de ressource d’investissement autonome, et de l’accompagnement des structures privées par le système public pourrait expliquer cette insuffisance. En résumé, les structures privées doivent être renforcées pour l’amélioration de cette sous-composante.

### Système d’information de la gestion logistique

Cette sous-composante a été appréciée « mauvaise » avec 47,30% du score attendu. Des points faibles du SIGL observés sont: la faible promptitude et complétude des rapports des FS et DRZ; une faible proportion de gestionnaires des ILP formés sur le remplissage des outils du SIGL (33,33%) avec pour corollaire des insuffisances dans le remplissage des fiches de gestion de stock. Cette situation prévalait aussi bien dans les FS publiques que privées et était d’autant plus important que les structures privées sont les plus nombreuses et très faiblement impliquées dans les activités de lutte contre le paludisme. Diagne et al. en 2012 [4] lors d’une évaluation rapide du Système pharmaceutique public en Guinée, avaient également identifié plusieurs points affectant la qualité du SIGL, notamment le manque de formation du personnel sur la gestion pharmaceutique et la faible promptitude et complétude des rapports. Par contre en 2006 au Rwanda, Ouédraogo et al. [12] avaient révélé que 100% des dépôts de districts et des FS utilisaient les formulaires logistiques (les fiches de gestion de stocks, support de rapport et bon de commande). Dans 58,33% de structures de notre étude, on notait une discordance entre les données du rapport et celles de l’inventaire physique au moment de l’élaboration des rapports ou commandes. Cette discordance variait entre 7,7% et 53,7% dans l’étude de la RDC [10]. Ce mauvais résultat du SIGL dans notre étude peut avoir plusieurs raisons: l’insuffisance de personnel, la faible implication des responsables des FS dans l’analyse périodique des supports et des données de gestion des intrants. Par ailleurs, les équipes de supervision ne prennent pas suffisamment de temps pour faire une analyse minutieuse des supports remplis dans les FS, il n’existe pas une surveillance rigoureuse de la transmission des rapports et il n’existe à aucun niveau de sanctions positives comme négatives dans le système. En résumé, l’étude met en relief des insuffisances majeures du SIGL. Des actions à tous les niveaux sont nécessaires pour espérer obtenir une amélioration.

### Quantification/estimation des besoins en intrants

La quantification des besoins en ILP se faisait par les structures elles-mêmes dans 77,78% sous la responsabilité des gestionnaires des ILP. Mais le processus a des défaillances liées à la faible disponibilité des données du SIGL pour la prise de décisions. En effet dans notre étude, seulement 47,67% des structures utilisaient les données du SIGL pour l’estimation des besoins. Contrairement aux observations de Adino et al. en 2011 à Addis-Abeba [13], les quantifications des besoins en produits contraceptifs se faisaient dans 87% des cas par le niveau supérieur. De même, au Ghana, parmi les causes de l´insuffisance du SGL figuraient la mauvaise quantification des besoins en intrants ainsi qu’une mauvaise planification des achats [14]. En somme, les insuffisances de quantification des besoins en intrants est la résultante de la faiblesse du SIGL.

### La gestion des stocks

Dans la présente étude, 63,89% des structures disposaient de 100% des fiches de gestion de stock des 12 ILP évalués. La complétude des fiches était bonne dans 47,22% des structures enquêtées. Selon Ouédraogo et al. [10], à l’exception d’un produit qui a une disponibilité de fiche de gestion de stock dans 87,5% des FS, tous les autres ont une disponibilité de 100% et dans toutes les FS enquêtées; 87,5% des intrants sélectionnés avaient leur fiche de stock tenue à jour au passage des équipes d’enquête. Les insuffisances notées dans la disponibilité des fiches de gestion de stocks et dans leur remplissage contribuent à la faiblesse du SIGL, avec pour conséquences la non-disponibilité des données de qualité pour les planifications.

### Disponibilité des intrants dans les formations sanitaires

Le jour de la visite, tous les DRZ et toutes les FS étaient en rupture d’au moins un intrant. La diversité des intrants, leur faible disponibilité au niveau des agences de la CAME, les insuffisances dans l’utilisation des outils de gestion par les FS, le manque de rigueur dans le suivi des consommations des intrants à tous les niveaux sont autant d’éléments susceptibles d’expliquer ces résultats. La faible disponibilité des intrants dans les agences de la CAME s’exprime par la non satisfaction des quantités et des différents types d’intrants commandés par les DRZ. Des auteurs ont diversement obtenu d’autres résultats. Ouédraogo et al en 2006 en RDC [10] ont trouvé qu’aucun dépôt de zone n’avait enregistré de rupture de stock de contraceptifs au cours des six derniers mois précédent leur enquête. Selon Adino [13], 37,2% des établissements étaient en rupture de stock d´au moins un produit de laboratoire au moment de la visite. Au Rwanda, la rupture la plus observée le jour de l’enquête concernait un produit contraceptif et était constatée dans 53,3% des structures [12]. Ce résultat était considéré comme une amélioration par rapport aux évaluations antérieures entre 2002 et 2004 dans ce pays. Par contre à la Centrale d’Achat des Médicaments Essentiel Génériques et des Consommables Médicaux du Burkina Faso de 2008 à 2011, Zalle [15] a trouvé que le taux global de rupture de 45 médicaments traceurs augmentait chaque année passant de 0,56% en 2008 à 4,63% en 2011. L’analyse des données de disponibilité des intrants montre qu’en dehors de la Quinine 600 mg injectable qui avait un taux de disponibilité de 75% dans les DRZ et 66% dans les FS, aucun autre médicament n’avait une disponibilité atteignant 50%. La plupart des FS privées n’étaient autorisées à s’approvisionner dans les agences de la CAME. Tout ceci constitue une porte ouverte au développement de circuits parallèles d’approvisionnement et l’introduction d’intrants hors la liste officielle dans leurs stocks. Les conséquences seront la persistance de la non maîtrise des consommations et les difficultés pour la quantification.

### Le niveau de performance du SGL des ILP dans le département du Littoral

Il se dégage des relations de cause à effet entre les résultats des différentes sous-composantes dans notre étude. En effet, l’insuffisance en nombre et en formation des ressources humaines chargées de la gestion des ILP, couplée à des insuffisances dans leur supervision peut avoir pour conséquences les faiblesses dans l’exécution des différentes fonctions du cycle logistique, notamment la mise en œuvre du SIGL. Ces faiblesses vont se répercuter dans la disponibilité des intrants. La non-conformité des magasins de stockage aura des effets négatifs sur la qualité des intrants à offrir aux bénéficiaires, l’ensemble engendrant une performance globale du SGL des ILP jugée insuffisante.

## Conclusion

La présente a révélé que la performance globale du SGL des ILP dans le département du Littoral en 2017 était « Insuffisante ». Des points faibles les plus importants identifiés portaient sur le SIGL, avec pour corollaires, la faible disponibilité des intrants ainsi que la faible disponibilité et utilisation des données du système pour les prises de décisions. Vu l’importance des intrants dans la lutte globale contre le paludisme, la mise en place dans les centres de santé y compris le secteur privé, d’une démarche qualité portant sur le SIGL ainsi que le développement d’un système de veille pour l’identification et la destruction des circuits parallèles d’approvisionnement et d’introduction d’intrants hors la liste officielle sont nécessaires.

### Etat des connaissances actuelles sur le sujet

- Il existe des dysfonctionnements dans la gestion des ILP au Bénin comme dans plusieurs autres pays en Afrique, entrainant des péremptions et des ruptures de stock paradoxales;
- Plusieurs acteurs sont impliqués dans la gestion des ILP dans le système, entre autres le PNLP, la centrale d’achat des médicaments essentiels, les partenaires techniques et financiers, les ZS, les FS aussi bien publiques que privées;
- Un circuit de l’information sur l’approvisionnement et l’utilisation des ILP est défini et diffusé au niveau des différents acteurs.

### Contribution de notre étude à la connaissance

- Cette étude démontre que la performance du SGL des ILP dans le département du Littoral au Bénin était « Insuffisante »; cette situation constitue un obstacle à la lutte efficace contre le paludisme;
- Le développement d'un SGL des ILP efficace pour la lutte contre le paludisme nécessite: la mise en place dans les centres de santé y compris le secteur privé, d’une démarche qualité dans la collecte et l’utilisation des données sur le système logistique de lutte contre le paludisme;
- Le développement d'un système de veille pour l’identification et la destruction des circuits parallèles d’approvisionnement et d’introduction d’intrants hors la liste officielle dans les stocks d’intrants de lutte contre le paludisme.

## Conflits d’intérêts

Les auteurs ne déclarent aucun conflit d'intérêts.
